# Peptides containing the PCNA interacting motif APIM bind to the β-clamp and inhibit bacterial growth and mutagenesis

**DOI:** 10.1093/nar/gkaa278

**Published:** 2020-04-29

**Authors:** Aina Nedal, Synnøve B Ræder, Bjørn Dalhus, Emily Helgesen, Rune J Forstrøm, Kim Lindland, Balagra K Sumabe, Jacob H Martinsen, Birthe B Kragelund, Kirsten Skarstad, Magnar Bjørås, Marit Otterlei

**Affiliations:** 1 Department of Clinical and Molecular Medicine, Faculty of Medicine and Health Sciences, Norwegian University of Science and Technology, NTNU, 7489 Trondheim, Norway; 2 Department of Medical Biochemistry, Institute for Clinical Medicine, Oslo University Hospital and University of Oslo, 0424 Oslo, Norway; 3 Department of Microbiology, Oslo University Hospital, and University of Oslo, 0424, Oslo, Norway; 4 Department of Biology, University of Copenhagen, Ole Maaloes Vej 5, 2200, Copenhagen N, Denmark

## Abstract

In the fight against antimicrobial resistance, the bacterial DNA sliding clamp, β-clamp, is a promising drug target for inhibition of DNA replication and translesion synthesis. The β-clamp and its eukaryotic homolog, PCNA, share a C-terminal hydrophobic pocket where all the DNA polymerases bind. Here we report that cell penetrating peptides containing the PCNA-interacting motif APIM (APIM-peptides) inhibit bacterial growth at low concentrations *in vitro*, and *in vivo* in a bacterial skin infection model in mice. Surface plasmon resonance analysis and computer modeling suggest that APIM bind to the hydrophobic pocket on the β-clamp, and accordingly, we find that APIM-peptides inhibit bacterial DNA replication. Interestingly, at sub-lethal concentrations, APIM-peptides have anti-mutagenic activities, and this activity is increased after SOS induction. Our results show that although the sequence homology between the β-clamp and PCNA are modest, the presence of similar polymerase binding pockets in the DNA clamps allows for binding of the eukaryotic binding motif APIM to the bacterial β-clamp. Importantly, because APIM-peptides display both anti-mutagenic and growth inhibitory properties, they may have clinical potential both in combination with other antibiotics and as single agents.

## INTRODUCTION

The DNA sliding clamps are functionally and structurally conserved in all three domains of life, although the sequence homologies are modest (1). Two interacting motifs are identified for the trimeric eukaryote DNA sliding clamp PCNA; the PCNA interacting peptide (PIP)-box (Q-xx-Ψ-xx-ϑϑ) (ϑ = aromatic, Ψ = hydrophobic, x = any residue) (2) and the AlkB homolog 2 PCNA Interacting Motif (APIM) (R/K- F/W/Y- L/I/V/A- L/I/V/A- K/R) (3). Whereas the PIP-box is found in many proteins essential for DNA replication, APIM is more frequently found in proteins important for handling of cellular stress, including proteins involved in DNA repair and translesion synthesis (TLS) (3–9). APIM and PIP-box have overlapping interaction sites on PCNA that both include the conserved hydrophobic pocket below the interdomain connecting loop (IDCL) (10,11). PCNA is a homotrimer, while the prokaryotic DNA sliding clamp, the β-clamp, is a homodimer and a subunit of the DNA polymerase III (Pol III) holoenzyme (12). The β-clamp is highly conserved between bacteria (13), and a loosely defined β-clamp binding motif of 5–6 amino acids (QL-x_1–2_-LF) (CBM), found in all prokaryotic DNA polymerases (14,15), is suggested to be the prokaryotic ancestor to the PIP-box. CBM interacts with the hydrophobic pocket at the C-terminal part of the β-clamp (16,17), similar to the APIM and PIP-box interactions with PCNA.

More than 600 mammalian proteins contain either APIM or the PIP-box, and can thus potentially interact with PCNA via a common interaction region (18). Binding to PCNA is therefore highly regulated by multi-layered mechanisms, which includes different post-translational modifications (PTMs) and large variations in binding affinities within and between the two interaction motifs (reviewed in (19,20)). For example, APIM-EYFP overexpression does not affect cell proliferation under unperturbed conditions, while PIP-box-EYFP overexpression in human and yeast cells is cytotoxic, likely due to inhibition of replication (3,21). APIM-EYFP overexpression, on the other hand, does not impair DNA replication but induces hypersensitivity toward chemotherapeutics, and APIM-EYFP is shown to pull down PTM variants of PCNA (3). PCNA modifications after cellular stress increase the affinity for APIM, e.g. poly-ubiquitination (22), and it is therefore suggested that mainly cellular stress defense mechanisms are impaired by APIM-peptides (10,23–26). It is not known if interactions between the β-clamp and different groups of protein-containing CBMs are similarly regulated.

The high-fidelity replicative polymerase III (Pol III) cannot replicate through bulky lesions in DNA. To prevent replication fork collapse and DNA double-strand breaks upon stalling of replication forks, cells utilize DNA damage tolerance mechanisms to bypass fork-stalling lesions. This lesion bypass occurs mainly after the re-initiation of new replications forks in a process where the replication fork skips the lesion and re-primes downstream of the lesion. The gaps left behind are filled using either the homolog DNA strand as a template or by utilizing specialized TLS polymerases which can polymerize over the lesions (reviewed in (27)). Because bypass by TLS polymerases often is done in an error-prone manner, these polymerases are important contributors to point mutations and thus essential in the development of drug resistance in both prokaryotes and eukaryotes (28–30). The catalytic subunit REV3L of the human polymerase POL ζ and the RAD5 homologs HLTF and SHPRH, all involved in TLS, were recently shown to have functional APIM PCNA interacting motifs, and targeting PCNA with APIM-peptides were shown to reduce the mutation frequency after UV-induced DNA damage in several cell lines (8,9). Direct interactions between the β-clamp and the prokaryotic TLS polymerases Pol II, Pol IV and Pol V, are shown to be vital for their TLS activity (31).

Pol II and Pol IV are expressed at low levels also in the absence of stress (30–50 and ∼250 copies per cell, respectively), but during SOS their expression is increased 7- to 10-fold. Pol V (UmuD’_2_C) on the other hand is almost completely SOS controlled, and functional Pol V is mainly detected after SOS-induction (32). It is believed that the concentration of the TLS polymerases, which are controlled by SOS, is the main trigger for TLS (reviewed in (27), but that low levels of constitutively expressed Pol II and Pol IV participate at stalled replication forks in absence of full SOS (33). The expression of mammalian TLS polymerases is more constant and not rapidly induced upon stress; however, their activities are tightly regulated to avoid excessive mutations. This includes regulation of their affinity for the DNA clamp, e.g. PCNA is mono-ubiquitinated upon stalling of replication and mono-ubiquitination is believed to be important in the regulation of the polymerase switch from the replicative polymerases to TLS polymerases in eukaryotic cells (reviewed in (34)). It is not shown that prokaryotes utilize the ubiquitin system for maintaining balanced systemic responses, but it is shown that they have several ubiquitin ligase enzymes that can hijack the ubiquitin system of various hosts (35). Prokaryotes also contain several ubiquitin-like peptide systems (36–38). However, whether PTMs, ubiquitin-like or others, are added to the β-clamp after stalling of the replications fork, and exactly how the switch from replicative polymerases to TLS polymerases is regulated in prokaryotes is elusive.

APIM-peptides interact with PCNA and are shown to affect DNA repair, TLS, apoptosis and cell signaling in mammalian cells, and to have anti-cancer efficacy in multiple preclinical animal models (4,8–10,18,23–26,39). A lead APIM-peptide, ATX-101, is currently in an ongoing Phase I clinical safety (maximal tolerated dose)-trial in cancer patients, initiated fall of 2018. Here, we show that APIM-peptides also have antibacterial activities both *in vitro* and *in vivo*. The APIM-peptides inhibit replication via interaction with the β-clamp, a promising drug target (40,41) (reviewed in (13,42)). Reduced mutation frequency after the addition of sub-lethal concentrations of the APIM-peptides indicates that the interactions between TLS polymerases and the β-clamp are inhibited by APIM-containing peptides at lower concentrations than the replicative polymerase. Because mutations introduced by TLS polymerases are important in the development of antimicrobial resistance (AMR), the anti-mutagenic activity of the APIM-peptides could have a large potential for use in combination with other antibiotics to prevent the development of resistance. In addition, the APIM-peptides efficiently inhibit bacterial growth at low concentrations in multidrug-resistant *Staphylococcus epidermidis* and *Staphylococcus aureus*.

## MATERIALS AND METHODS

### Species, strains, materials, growth measurements

*S. aureus:* ATCC 29213, FDA486, methicillin-resistant *S. aureus* strain (MRSA) 43484 and FusA88779; *S. epidermidis:* clinical isolates (including one gentamicin sensitive strain and several multidrug-resistant strains) from Department of Microbiology, St. Olavs University Hospital, Trondheim, Norway, kindly provided by professor Kåre Bergh; *Escherichia coli*: strains DH5α, BL21 (RIPL) DE3 (Novagen), K12 (MG1655) wild type (WT) and TLS deletion strains (ΔPol II^3^, ΔPol IV^1^, ΔPol V^1^, ΔPol II/IV^3^, ΔPol II/V^3^, ΔPol IV/V^2^ and ΔPol II/IV/V^3^). ^1^ Keio collection, ^2^ generated by Prof Magnar Bjørås’ group, UiO and ^3^prepared as described (43). Briefly, *polB* was inactivated in *E. coli* MG1655 WT, ΔPol IV, ΔPol V and ΔPol IV/V using JW0059:: *polB*. A phage stock (T4GT7) diluted in T4 buffer was added to the JW0059::*polB* culture, incubated and plated on LB agar plates. The lysate was harvested and added to 2 ml T4 buffer and 0.2 ml CHCl_3_ (T4GT7 lysate). The mixture was centrifuged, and supernatant stored at 4°C over 20 μl CHCl_3_. 0.5 ml overnight culture of the recipient cells was re-suspended in 1 ml of T4 buffer and aliquoted. The T4GT7 lysate (10^−1^, 10^−2^, 10^−3^ and 10^−4^ dilutions) was added to the aliquots, incubated for 25 minutes (min) at room temperature and plated on LB-agar plates with kanamycin. Colonies obtained were re-streaked twice to obtain a pure culture. Kanamycin resistance cassettes replacing the deleted *polB* genes were removed by an FLP-recombinase expressed by pCP20 plasmid.

Verification of the deletions strains was done by polymerase chain reaction (PCR) using the following primers:

Δ Pol II strain:  5′-GATTCCAGCAACCGGCACCAC-3′ and 5′-CAGGCGAGTACCGATATAGG-3′
Δ Pol IV strain:  5′-TATGTTTGAACGCGGCAGCG-3′ and 5′-AGTCATTGAAATCATCATCC-3′
Δ Pol V strain:  5′-TGTTTAATGTCATTATGGCG-3′ and 5′-AAGTGTTTGTCGCCTCTGGC-3′

### Minimal inhibitory concentration assay

Minimal inhibitory concentration (MIC) was determined following the protocol recommended by the National Committee of Laboratory Safety and Standards (NCCLS, (44)) for microtiter broth dilution assay, conducted with modifications suggested by R.E.W. Hancock Lab for cationic peptides (45). Briefly, bacterial suspension in Mueller-Hinton Broth was adjusted to 0.5 McFarland and diluted to 5 × 10^5^ CFU/ml. The suspension (0.1 ml) was seeded in polypropylene microtiter plates (Greiner), 5 × 10^4^ CFU/well. APIM-peptides were added as 10% of the total volume. Initial inoculums were quantified by seeding on agar for verification of the estimated CFU/mL. After 24 hours (h) incubation at 37°C, the microtiter plates were inspected for visible growth.

Determination of minimal inhibitory concentrations (MICs) for *S. aureus* was done at Statens Serum Institute, Copenhagen, Denmark.

### Cultivation

Growth experiments were performed in Luria-Bertani broth (LB) media, Tryptic soy broth (TSB) or LB agar at 37°C, antibiotic selections were performed using the following concentrations: kanamycin 30 μg/ml, chloramphenicol 34 μg/ml and rifampicin 100 μg/ml.

### Peptides

Table 1 shows an overview of the peptides (Innovagen, Sweden) used.

**Table 1.** Peptides used in the different experiments

Cell penetrating peptides	Sequence	Used in experiments
RWLVK	Ac-MD-RWLVK-WKKKRKI-RRRRRRRRRRR	MIC-assay, Rif^R^ assay, MST
RWLVK*	Ac-MD-RWLVK-GILQWRKI-RRRRRRRRRRR	MIC-assay, DNA replication assay, Rif^R^ assay
RFSLK	Ac-MD-RFSLK-WKKKRKI-RRRRRRRRRRR	MIC-assay, Mutation frequency (Gen^R^) assay, Rif^R^ assay, *In vivo* skin model, MST
RFSLK*	Ac-MD-RFSLK-GILQWRKI-RRRRRRRRRRR	MIC-assay
RALVK	Ac-MD-RALVK-WKKKRKI-RRRRRRRRRRR	MIC-assay, Rif^R^ assay, MST
RWLK	Ac-MD-RWLK-WKKKRKI-RRRRRRRRRRR	MIC-assay, Rif^R^ assay, MST
R11	RRRRRRRRRRR	MIC-assay, DNA replication assay, Rif^R^ assay, MST
**Overexpressed** **peptides**		
RWLVK	MD-RWLVK- GILQSTLA	Rif^R^ assay, Overexpression (viability) assay
RFSLK	MD-RFSLK- GILQSTLA	Rif^R^ assay, Overexpression (viability) assay
RALVK	MD-RALVK- GILQSTLA	Overexpression (viability) assay
RWLK	MD-RWLK- GILQSTLA	Rif^R^ assay, Overexpression (viability) assay
**Octamer** **peptides**		
RWLVK	Ac-MDRWLVKW	SPR analysis, NMR spectroscopy
RALVK	Ac-MDAWLVKW	SPR analysis, NMR spectroscopy
Pol III peptide	Ac-EQVELEFD	NMR spectroscopy

* = different linker between APIM motif and cell penetrating part (R11). The C-termini on all peptides were amidated. All peptides were TFA-salt (>90% pure). Lyophilized peptide from the manufacturer was dissolved in stock solutions of 1 mM peptide in water.

### Cloning and overexpression

Oligonucleotides (Sigma) for annealing and cloning of **APIMs** into the *Nco*I/*Nhe*I site of pET28a(+) (Novagen):

**Table:** 

RWLVK:	5′-CATGGACAGATGGCTGGTGAAAGGAATTCTGCAGTCGACG-3′ and
	5′-CTAGCGTCGACTGCAGAATTCCTTTCACCAGCCATCTGTCC-3′,
RFSLK:	5′-CATGGACAGATTTAGTCTGAAAGGAATTCTGCAGTCGACG-3′ and
	5′-CTAGCGTCGACTGCAGAATTCCTTTCAGACTAAATCTGTCC-3′,
RFLSK:	5′-CATGGACAGATTTCTGAGTAAAGGAATTCTGCAGTCGACG-3′ and
	5′-CTAGCGTCGACTGCAGAATTCCTTTACTCAGAAATCTGTCC-3′,
RALVK:	5′-CATGGACAGAGCTCTGGTGAAAGGAATTCTGCAGTCGACG-3′ and
	5′-CTAGCGTCGACTGCAGAATTCCTTTCACCAGAGCTCTGTCC-3′,
RWLK:	5′-CATGGACAGATGGCTGAAAGGAATTCTGCAGTCGACG-3′ and
	5′-CTAGCGTCGACTGCAGAATTCCTTTCAGCCATCTGTCC-3′.

A stop codon is found in the multiple cloning site after the *Nco*I/*Nhe*I site. The sequence over-expressed is MD-**APIM**-GILQSTLA.

### APIM-EYFP constructs

APIM-sequences (RWLVK and RWLK) were fused with EYFP in the pEYFP vector (Clonetech) and were cloned into pET28a(+) using the NcoI/NheI sites. Peptide variants (RFSLK and RALVK) were made by site-directed mutagenesis by GenScript. All constructs were confirmed by sequencing performed by GATC Biotech. Cloning was performed in *E. coli* DH5α. Expression levels of APIM-EYFP were measured by western analysis. Briefly, cell pellets were boiled in OmniCleave™ buffer, followed by incubation with OmniCleave™ endonuclease (200 U). Samples were next added lithium dodecyl sulphate (LDS) loading buffer (Invitrogen, Thermo Fisher Scientific) containing 100 mM DTT and incubated 10 min at 70°C before loaded on a 4–12% polyacrylamide gel (NuPAGE™, Invitrogen). Proteins were blotted to a PVDF membrane (Immobilon, 0.2 μM), blocked in 5% bovine serum albumin. Antibodies utilized were αGFP (Ab290, Abcam) and IRDye^®^ 680RD goat α-rabbit (LI-COR).

### Overexpression experiments in *E. coli* BL21

Induction was performed using isopropyl β-D-1-thiogalactopyranoside (IPTG) (1 mM) at OD_600_ = 0.3 - 0.4. Bacterial growth was measured by OD_600_ or the number of colony-forming units (CFU) per ml.

### Replication assay

*E. coli* MG1655 cells were grown at 37°C in glucose-CAA medium (AB minimal medium (46) supplemented with 10 μg/ml thiamine, 0.2% glucose and 0.5% casamino acids) for several generations to ensure balanced growth. At an OD_600_ = 0.15, the cells were added 2, 4 or 6 μM RWLVK* peptide or 2, 4, 6 or 15 μM R11 peptide. Growth was continued for 2–5 min. After peptide treatment, cells were either harvested directly or treated with rifampicin (Rif) (300 μg/ml) and cephalexin (Cpx) (10 μg/ml) for the time equivalent of three to four generations before harvesting. Both directly harvested and Rif and Cpx treated cells were re-suspended in TE buffer (10 mM Tris–HCl, pH 8.0, 1 mM EDTA, pH 8.0) and fixed in 70% ethanol. Fluorescein isothiocyanate (FITC, Sigma-Aldrich) (47) was used for protein staining (representing mass), and Hoechst 33258 was used for DNA staining (Sigma-Aldrich) (48). Flow cytometry was performed with an LSR-II flow cytometer equipped with a 488 nm argon-ion laser and a 355 nm krypton laser (BD Biosciences), and the results were analyzed using FlowJo software (Tree Star, Inc.). Rif and Cpx inhibit the initiation of replication and cell division, respectively. Cells cannot initiate a new round of replication after these drugs are added but can complete ongoing rounds of replication (without dividing). Therefore, DNA histograms of the Rif and Cpx treated cells give an integer number of chromosomes per cell with values 2^n^ or 2^n+1^, where *n* = 0, 1, 2, 3… represents the generation in which initiation occurs. However, this pattern holds true only for cells with successful completion of replication elongation (all initiated replication forks reaching the terminus). Problems with replication elongation will be seen as a lack of distinct peaks in the ‘run-out’ DNA histograms (i.e. histograms of cells treated with Rif and Cpx).

### Site-directed mutagenesis of the β-clamp

The plasmid containing the *dnaN* gene encoding β-clamp, pET-16b, was isolated from *E. coli* BL21. Site-directed mutagenesis was performed following the Stratagene QuikChange^®^ Site-Directed Mutagenesis Kit protocol (Revision A, 2006). The single residues were mutated using the following oligonucleotides (Sigma):

**Table:** 

H148K	5′-CAGTTTTCTATGGCGAAACAGGACGTTCGCTATTAC-3′
	5′-GTAATAGCGAACGTCCTGTTTCGCCATAGAAAACTG-3′
V151A	5′-GGCGCATCAGGACGCTCGCTATTACTTAAATGG-3′
	5′-CCATTTAAGTAATAGCGAGCGTCCTGATGCGCC-3′
P242D	5′-GGTCGCTTCGACGATTATCGCCGCGTTC-3′
	5′-GAACGCGGCGATAATCGTCGAAGCGACC-3′
V247F	5′-GGATTATCGCCGCTTTCTGCCGAAGAACCC-3′
	5′-GGGTTCTTCGGCAGAAAGCGGCGATAATCC-3′
M362D	5′-GCGGCTTATGTTGTCGATCCAATGAGACTG-3′
	5′-CAGTCTCATTGGATCGACAACATAAGCCGC-3′
P363D	5′-GCGGCTTATGTTGTCATGGACATGAGACTG-3′
	5′-CAGTCTCATGTCCATGACAACATAAGCCGC-3′

### Purification of β-clamp protein

*E. coli* BL21 cultures were grown and induced for protein expression of His-tagged WT and mutant β-clamp from pET-16b (including *dnaN* gene*)* with 300 μM IPTG at OD_600_ = 0.1–0.2. The bacterial cultures were centrifuged 2 h after induction with IPTG and pellets were lysed with lysis buffer containing 1 mg/ml lysozyme (Sigma-Aldrich) and sonicated (Branson Sonifier 250). The His-tagged β-clamp was purified using a TALON^®^ superflow™ metal affinity resin (Clonetech Laboratories Inc.) and NaP-buffers (50 mM, pH 8) supplemented with 300 mM NaCl, 0.01% tween and 10 mM β-mercaptoethanol, followed by imidazole elution (150 mM). To concentrate the proteins the elution buffer was run through Amicon® Ultra centrifugal filters (Ultracel® 10 K, Merck Millipore). The proteins in the centrifuge filter were washed out with phosphate-buffered saline (PBS) and finally added glycerol (50%) for storage. For nuclear magnetic resonance (NMR) spectroscopy, the pellet was resuspended in lysis buffer (20 mM Tris, 100 mM NaCl, 2.5 mM MgCl_2_, 0.5 mM CaCl_2_, 10 μg/μl DNase 1, pH 7.4) and lysed by French Press at 20 KPSI and subsequently centrifuged at 20 000 *g* for 30 min at 4°C. The supernatant was loaded onto a 5 ml HisTrap FF pre-equilibrated in binding buffer (20 mM Tris, 100 mM NaCl pH 7.4) and afterward washed with five column volume (CV) wash buffer (20 mM Tris, 100 mM NaCl, 10 mM Imidazole pH 7.4). The protein was eluted with 2 CV elution buffer (20 mM Tris, 100 mM NaCl, 250 mM imidazole pH 7.4) and loaded onto a size exclusion chromatography column (HiLoad 16/600 Superdex 200 pg) for further purification using 20 mM NaH_2_PO_4_, 100 mM NaCl, pH 7.4.

### β-clamp binding assays

#### Microscale thermophoresis (MST)

Purified *E.coli* β-clamp (49) was labeled using the RED-Maleimide Labelling Kit (NanoTemper Technologies, GmbH) according to the manufacturer's instructions applying a concentration of 20 μM protein at room temperature. The ligands, cell penetrating APIM-peptides, were dissolved and diluted in supplemented Microscale thermophoresis (MST) buffer (50 mM Tris–HCl pH 7.5, 150 mM NaCl, 10 mM MgCl_2_) with 2% fetal bovine serum and incubated for 10 min in room temperature. Series of 1:1 dilutions (16×), producing ligand concentrations ranging from 500 μM to 15 nM, were made. Each ligand solution was further diluted 1:1 with fluorescent labeled β-clamp (diluted in MST buffer, 3 nM) and incubated for 10 min. Fluorescence was measured for each protein-ligand sample using a Monolith NT.115 instrument (20% MST power and 100% LED power) and Monolith NT Standard Treated Capillaries (both NanoTemper Technologies, GmbH). To calculate the *K*_D_’s, fluorescence signals were plotted and analyzed using the supplemented software (NT Analysis software version 1.5.41 and MO.affinity analysis version 2.2.4, NanoTemper Technologies). Denaturation with SD-buffer (4% sodium dodecyl sulphate and 40 mM DTT) and re-measurement of total fluorescence confirmed that the observed change in fluorescence was due to ligand binding event.

#### NMR spectroscopy

Each octamer peptide (MDRWLVKW, MDRALVKW or Pol III β-clamp binding peptide EQVELEFD, APIM and potential CBM underlined) was added sub-stoichiometric amounts of β-clamp depending on the expected affinities (100 or 50 μM peptide added 5 or 20 μM β-clamp, respectively). All NMR spectra were recorded in a standard 5 mm NMR tube at 298 K in 20 mM NaH_2_PO_4_, 100 mM NaCl, pH 7.4, 0.25 mM DSS, 10% D_2_O on a Bruker AVANCE III 800-MHz (^1^H) spectrometer equipped with a room temperature probe. Free induction decays were transformed and analyzed in Topspin (Bruker Biospin) and the aromatic region assigned.

#### Surface plasmon resonance (SPR) analysis

The experiment was performed with Biacore S200 (GE Healthcare), at 25°C with a flowrate of 30 μl/min. Purified WT and mutant β-clamp protein were immobilized by amine coupling on CM5 Series S sensor chips (GE Healthcare). The proteins were immobilized in 10 mM sodium acetate buffer with pH 4.0 until a level of approximately 2000 response units (RU) were reached in flow channel number two or four, leaving flow channel number one or three as reference channels.

Octamer peptides containing APIM motifs (underlined) MDRWLVKW and MDRALVKW were diluted in running buffer (PBS P+ buffer: 20 mM phosphate buffer pH 7.4, 3 mM KCl, 137 mM NaCl, 0.05% surfactant P20) at 4, 8, 16, 32, 64 and 128 μM, and analyzed by single-cycle mode. Six consecutive injections of alternating running buffer or peptide in running buffer, with an association phase of 120 seconds (s) followed by a dissociation phase of 60 s and a final dissociation phase of 600 s, were run for each β-clamp protein variant. The running buffer series were used as blanks together with the signal from the reference channels for double-reference subtraction. After each series of six injections, the sensor chip was washed with a 120 s regeneration injection of 1 M NaCl followed by a 120 s injection of running buffer. The sensorgrams were processed (reference subtracted) with Biacore S200 Evaluation Software Version 1.1 (GE Healthcare) and normalized according to the immobilized level. Data were exported to Origin 2017 (OriginLab) for the preparation of figures.

#### Mutagenesis assays

The rifampicin resistance (Rif^R^) mutagenicity assay was performed as described in (50) with some modifications. Briefly, *E. coli* BL21 expressing peptide variants were induced with (IPTG) (1 mM) at OD_600_ = 0.3–0.4 and cultured at 37°C, for 2 h before harvested. Bacteria from large culture volumes (up to 50 ml) were plated in order to obtain ∼100 Rif^R^ colonies per dish. The anti-mutagenic effect of cell penetrating APIM-peptides in *E. coli* MG1655 was done by adding 20 μM peptide at OD_600_ = 0.01, and the cells were cultured for 30 min. Next, the cells were pelleted and washed with PBS before UV-irradiated in (50 μl) PBS (2 mJ UVC/cm^2^), re-suspended in 2.5 ml LB and cultured in 37°C for 2 h before plating on LB plates with and without Rif (100 μg/ml). The number of Rif^R^ colonies/ total CFU was determined after 1 and 2 days, respectively. Significance was calculated by using a two-tailed, paired, students *t*-test. Gentamicin resistance (Gen^R^) was examined using a gentamicin sensitive clinical isolate of *S. epidermidis*. The overnight culture was diluted (1:100) into 4 ml TSB with an addition of ∼50% MIC of gentamicin (0.25 μg/ml), APIM-peptide (3 μM), a combination of these or a corresponding amount of water as a control. The cultures were grown in a shaking incubator (37°C) and were re-inoculated (1:100) every 24 h in fresh TSB and supplements. The cultures were plated on blood agar plates with and without gentamicin (10 mg/l) every day for 4 days, and the frequency of Gen^R^ colonies was determined by calculating the number of Gen^R^ colonies/total CFU.

#### Structural modeling

Atomic coordinates of β-clamp in complex with a peptide from DNA polymerase V (PDB code: 4k74, (17)) were retrieved and the residues QLNLF in the Pol V peptide were replaced with the residues MDRWL from the APIM motif, based on a sequence alignment where hydrophobic residues WL in the APIM motif are aligned with the corresponding hydrophobic LF motif in Pol V CBM. There are no residues in the template peptide to model the last two residues (VK) in the APIM motif. With this alignment, the key residue W in APIM can point into the hydrophobic pocket formed by, among others, residues V247 and M362 in the clamp. This initial model was used as an input for the Rosetta FlexPepDock protocol for high-resolution modeling of peptide-protein interactions (51) to generate alternative models for the interaction.

#### Murine skin wound infection model

The experiment was performed at Microbiology and Infection Control, Statens Serum Institute, Copenhagen, Denmark, approved by the National Committee of Animal Ethics, Denmark. The experimental procedure is modified from a previously described skin infection model in mice (52). A total of 32 Balb/C female mice (18–22 g, Taconic, Denmark) were used for the experiment. The mice were treated orally with analgesia, 45 μl Nurofen^®^junior (Novartis 20 mg/ml ∼30 mg/kg), 30–60 min before the experiment. The mice were anesthetized with 0.15 ml diluted Zoletil mix (2 ml Zoletil mix + 5.2 ml sterile saline + 2.8 ml Torbugesix 1:100) before the fur was removed on a 2 × 3 cm area of the back with an electric shaver and a razor. At last, a skin lesion was made on each mouse with a dermal curette (Miltex 335710). A total of 10 μl inoculum containing 10^7^ CFU MRSA 43484 (in saline) was added to a 1 cm^2^ area of the skin lesion of each mouse. One group of animals (six mice) were sacrificed 1 day after inoculation, before any treatment was given, to quantify the inoculum. The remaining mice were given topical treatment twice a day (9 a.m and 3 p.m) for 3 days; APIM-peptide (RFSLK, Innovagen SE, 12.8 mg/ml in 1% methyl cellulose gel containing 10% glycerol) or Fusidic acid 2% ointment (LeoPharma). A volume of 50 μl was used for each treatment, spread out on the skin lesion. One group of mice did not get any treatment and represent the control group. The treated animals and the control group were sacrificed on day 4, one day after the last treatment. The skin lesion area was removed by a pair of tweezers and scissors and collected in 1 ml saline before being homogenized in a Dispomixer. Each sample was serially diluted in saline/Triton-X and 20 μl spots were applied on Mueller Hinton/PolymyxinB 3% NaCl plates and incubated 24–48 h at 35°C. The mice did not show any clinical signs of systemic infection or distress.

## RESULTS AND DISCUSSION

### APIM-peptides have growth inhibitory activity in several bacterial species

During an accidental bacterial infection in our mammalian cancer cell cultures, we observed that in wells where APIM-peptide was added, the cell media looked normal, while in parallel wells without APIM-peptide, the cultures were overgrown with bacteria. At low APIM-peptide concentrations the cancer cells were growing normally; however, no bacterial growth was seen, indicating that the APIM-peptide had antibacterial activity at concentrations not inhibiting cancer cell proliferation (data not shown). It is well known that cationic peptides may have broad-spectrum antimicrobial activities, and this includes both synthetic peptides, such as cell penetrating peptides, and peptides synthesized by bacteria or eukaryotes (53). Much of the antibacterial effects of these cationic peptides are believed to be caused by disruption of the bacterial cell membrane. However, because PCNA and β-clamp share structural features, the antibacterial activity observed could possibly also be mediated by the PCNA interacting motif, APIM, which may interact with the β-clamp and thereby prevent bacterial replication.

**Figure 1.**
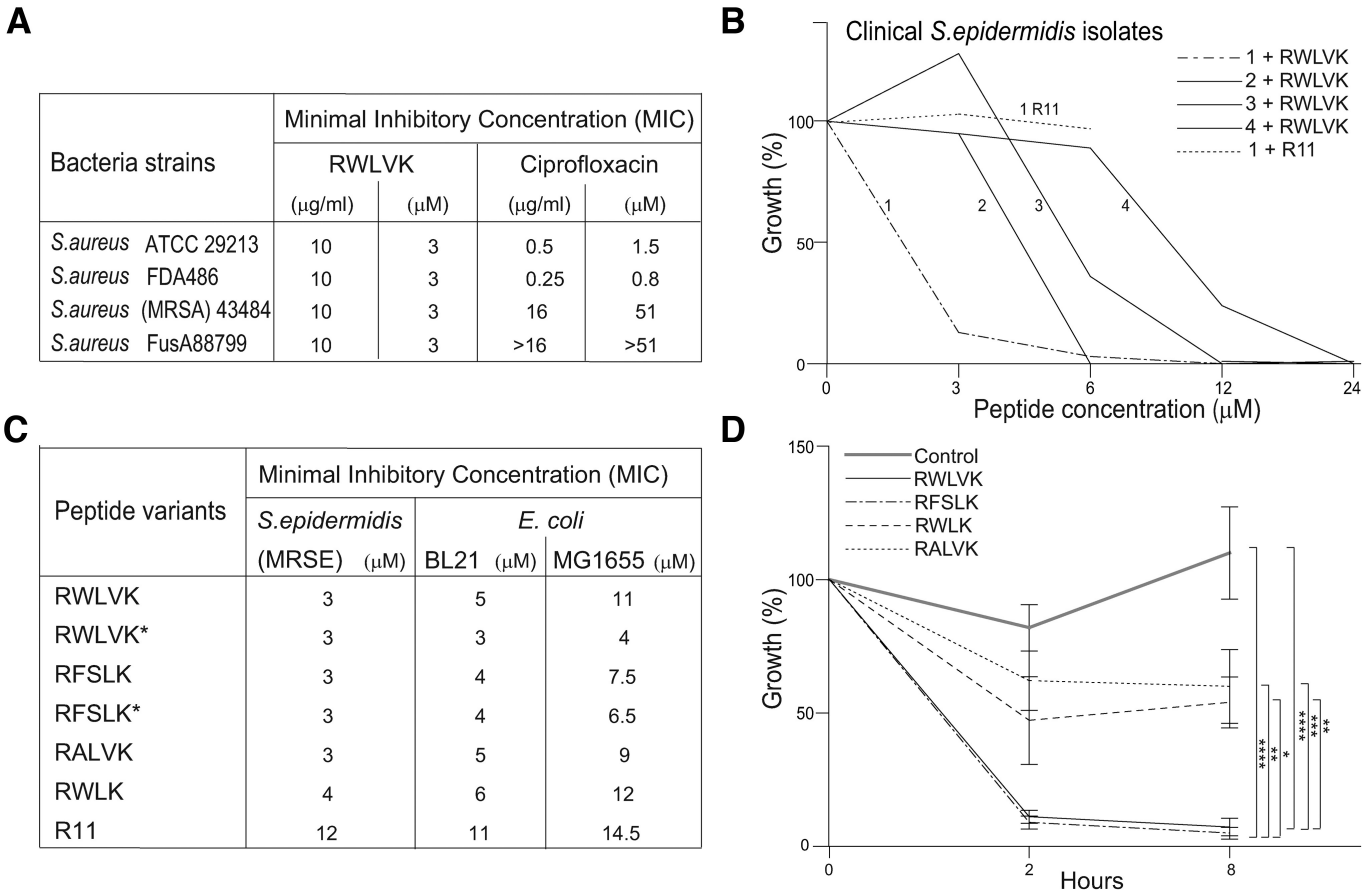
Cell penetrating peptides containing APIM (APIM-peptides) have growth inhibitory activities. (**A**) MIC determination (μg/ml and μM) for the cell penetrating APIM-peptide RWLVK (MW = 3674 Da) in different strains of *S*.*aureus*. MICs for ciprofloxacin (MW = 313 Da) for the strains determined in parallel experiments are given as a reference. The same results were obtained in three biological replicates (*n* = 3). (**B**) Inhibition of growth (OD_600_) of four different clinical isolates of multidrug-resistant *S. epidermidis* after the addition of the cell penetrating RWLVK-peptide. Data are given as % of untreated parallel of the same isolate. (**C**) MIC determination of *S. epidermidis* (MRSE), and two *E. coli* strains (BL21 and MG1655) for different cell penetrating APIM-peptide variants (connected to R11) or the cell penetrating peptide only (R11). Peptides marked with an asterisk (*) are peptides connected to R11 with linker ‘GILQ-WRKI’ instead of ‘W-KKKRK-I’. MIC-values are average of ≥3 independent biological experiments. (**D**) The effect of expressing different APIM-peptide variants without the cell penetrating peptide in *E. coli* BL21. The expression was induced with IPTG (1 mM) at ‘0 h’ in exponentially growing cultures. Growth (CFU/ml) 2 and 8 h after induction is displayed relative to its own uninduced culture (%). ‘Control’ culture = BL21 cells. Data presented represent average data from three technical replicas from ≥3 independent biological experiments (≥2 different bacteria clones for each peptide variant). *P* < 0.0001****, *P* < 0.001***, *P* < 0.01**, *P* < 0.05*, two-way ANOVA.

To explore the antibacterial activity of peptides containing APIM further, activities of cell penetrating peptides containing different APIM-consensuses were examined in both Gram negative and positive bacterial species. Low MICs were detected in both multidrug resistant *S. epidermidis* and *S. aureus* strains and in the laboratory *E. coli* strains BL21 and MG1655 with the RWLVK and RFSLK peptide variants (Figure 1A and C). For two of the resistant *S. aureus* strains, the RWLVK-peptide was much more efficient than the corresponding molar concentration of Ciprofloxacin (Figure 1A). The antibacterial activity varied between different bacterial species, and between different isolates of bacteria within the same species, as shown for different clinical isolates of multidrug-resistant *S. epidermidis* (Figure 1B). Why some clinical isolates of *S. epidermidis* are more sensitive to the APIM-peptide than others is not known; however, it could be speculated that mutations leading to loss of fitness are more pronounced in the most sensitive isolates. Different sensitivity to APIM-peptides between different bacterial species could also be due to difference in the bacteria's ability to import and digest the peptide (i.e. peptide stability), and/or differences between the β-clamps even though they have high structural homology (54). Differences in sensitivity toward the APIM-peptide are also seen between different cancer cell lines (10,39). Because cell penetrating peptides often have antibacterial activity on their own, we included a peptide containing only the cell penetrating part of the APIM-peptide, i.e. R11 (11 arginine residues). We found no effect of this peptide on the most sensitive of the clinical isolates of *S. epidermidis* at the same molar concentration as the full-length cell penetrating RWLVK-peptide (Figure 1B, compare line marked 1 R11 with the line marked 1). Furthermore, when comparing different APIM-peptide variants, MICs were lowest for the variants named RFSLK* and RWLVK*, and highest for the truncated APIM (RWLK) and the peptide containing only the cell penetrating part (R11) in both *E. coli* and *S. epidermidis* (Figure 1C). These results indicate that the full-length peptides containing a 5-residue APIM have strongest antibacterial activity. To examine if this additional growth inhibition were due to interactions of APIM with an intracellular target, possibly the β-clamp, we overexpressed short peptides containing the APIM-consensus in *E. coli* BL21. Overexpression of RWLVK and RFSLK strongly inhibited bacterial growth, while overexpression of a mutated APIM, RALVK, which is previously shown to have reduced affinity for PCNA both *in vivo* and *in vitro* (3,10,26), and the truncated version of APIM, RWLK, did not inhibit bacterial growth (CFU determination in Figure 1D, OD measurements in Supplementary 1A). The expression levels for the same APIM-versions fused N-terminally to EYFP were equal, suggesting that the difference in growth-inhibiting potential was not due to differences in peptide expression (Supplementary Figure S1B).

### APIM-peptides interact with the β-clamp

**Figure 2.**
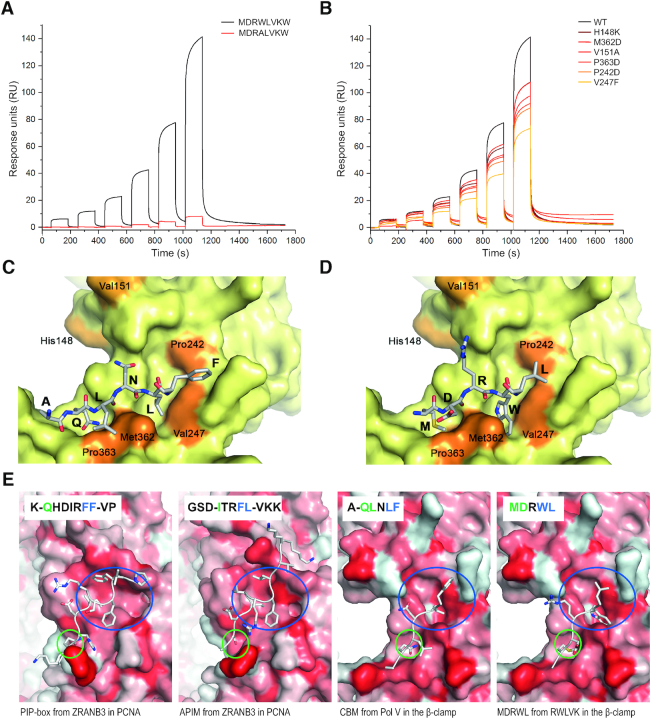
APIM-peptide variant RWLVK interacts with the β-clamp. (**A**) SPR analysis of immobilized WT β-clamp and the octamer peptide containing APIM, MDRWLVKW and mutated APIM, MDRALVKW. (**B**) SPR analysis of immobilized mutant β-clamps (H148K, M362D, V151A, P363D, P242D, V247F) and the MDRWLVKW-peptide. (**C** and **D**) Structural modeling of peptide interactions with the β-clamp (C) CBM from Pol V (UmuC), AQLNLF, (from (17)), and (D) part of the APIM-peptide, MDRWL. Mutated residues, used in (B), are highlighted in orange. Underlined amino acids are part of APIM or CBM consensus sequences. (**E**) Structure of PIP-box and APIM in ZRANB3 bound to PCNA (11,58), CBM from Pol V bound to the β-clamp (17) and a homology model of the MDRWL-part of the APIM-peptide based on alignment with the QLNL-part of the Pol V CBM-peptide. Blue and green circles illustrate the hydrophobic pocket/subsite I and Q-pocket/subsite II, respectively. Residues interacting with these pockets are shown with blue and green color in the sequence display (inset), respectively. The protein surfaces are colour coded from white to red according to the hydrophobicity index (66).

Initial *in vitro* binding experiments by MST indicated that cell penetrating versions of the RWLVK and RFSLK- peptides had higher affinities (*K*_D_s < 50 μM) for the β-clamp than the corresponding RALVK-peptide (*K*_D_ > 750 μM), and that no binding was detected for the truncated APIM-peptide RWLK and R11 (Supplementary Figure S2A). Next, we examined the direct interaction between the β-clamp and octamer peptides containing RWLVK and RALVK (i.e. without the cell penetrating part of the peptide) by SPR. Similar to what was indicated by the initial fluorescence measurements, the peptide containing RWLVK bound stronger to the β-clamp than RALVK (Figure 2A), which is in accordance with what is previously seen for the interaction of RWLVK and RALVK with PCNA (26). We further analyzed the binding of the same octamer peptides to the β-clamp using 1D ^1^H NMR spectroscopy (Supplementary 2B). Here, we included a positive control octamer peptide containing the β-clamp binding peptide from the disordered far C-terminal end of Pol III, ^1153^EQVELEFD^1160^, with a *K*_D_ of ∼2 μM (41) containing a CBM consensus sequence (Supplementary 2B). From the *K*_D_s, we expected the RWLVK-peptide to be in fast exchange between free and bound state on the NMR time scale, which would result in a visual line broadening effect already at a peptide: β-clamp ratio of 100:5, whereas for Pol III β-clamp binding peptide, we would expect slower exchange with loss of signal from the bound state. To be able to see this, we analyzed the Pol III β-clamp binding peptide at a ratio of 50:20. Indeed, visual line broadening was seen for RWLVK already at 100:5 and a similar effect was seen for the β-clamp binding peptide from Pol III at a ratio of 50:20. No chemical shift- or intensity changes were seen for the addition of the β-clamp to the RALVK, showing that at these concentrations we could not detect any binding of this peptide to the β-clamp by NMR. Thus, the RWLVK, but not the RALVK peptide bound directly to the β-clamp in these experiments, as did the peptide from Pol III.

To determine where the peptides bound on the β-clamp, mutations in the region previously shown to interact with CBMs of the polymerases were prepared (mutated residues: H148K, M362D, V151A, P363D, P242D, V247F, shown in orange in Figure 2C and D). Mutation of P363 of the β-clamp (P363S) has previously been shown to severely impair interaction with both Pol V (UmuC) and Pol III (α subunit) (16), in addition to impair Pol V dependent mutagenesis (55). P363 is directly interacting with peptides containing CBM from Pol V (UmuC) (shown in Figure 2C), in addition to peptides containing the β-clamp binding peptide ^1153^EQVELEFD^1160^ from Pol III (17). The more distant residues 148–152 form a loop in the β-clamp which is important for interaction with DNA in addition to interactions with all the TLS polymerases, even though the different TLS polymerases interact with this loop differently (56,57). All the variants of the β-clamp were found to have reduced ability to bind RWLVK, with the β-clampV247F being most affected with ∼50% reduced binding (Figure 2B).

The DNA-clamps from all three kingdoms of life share structural properties, such as the presence of a peptide-binding groove in the C-terminal region essential for interactions with the clamp binding motifs (reviewed in (1), and illustrated for the β-clamp and PCNA in Figure 2E). The five–six amino acids of CBM (QL-x_1–2_-LF) and what is believed to be its successor, the PIP-box (Q-xx-M/L/I-xx-FF/Y), share some similarities, while less similarity is found between CBM and APIM (R/K-F/Y/W-L/I/V/A-L/I/V/A-R/K). However, despite low similarity between APIM and PIP-box, it is shown that they have overlapping interaction site on PCNA (11,58). Analysis have shown that F_2_, L_3_ and K_5_ in the APIM sequence of ZRANB3 (**S**D**I**TR**FL**V**K,** APIM underlined) binds in the hydrophobic pocket, also called the ‘socket’, of PCNA, forming a hydrophobic plug, and that an S- and I- residue four and two residues upstream of APIM, respectively, bind to the Q-pocket of PCNA (58). The corresponding amino acids in the PIP-box in ZRANB3 (**Q**HD**I**RS**FF**) are FF and I binding in the socket and Q in the Q-pocket (Figure 2E). The socket in PCNA corresponds to interaction subsite I on the β-clamp, and the Q-pocket is often referred to as subsite II (41). It is suggested that the pair of hydrophobic residues at positions four/five or five/six in CBM (i.e. L_4_F_5_ for Pol V) comprise the core of CBM and that these interact with subsite I on the β-clamp (14). Based on the resemblance in structure between the β-clamp and PCNA, and the known co-structure of the Pol V (UmuC) CBM, QLNLF and the β-clamp (17), we suggest that WL in the APIM-peptide (MDRWLVK) bind in subsite I and MD in subsite II (Figure 2D and E). In this homology model, W4, forms interactions with V247 and/or M362. A peptide docking of this initial model using the Rosetta FlexPepDock protocol suggests that the W-residue might interact with subsite I in two different orientations (Supplementary Figure S3A–E). Experimental data is required to verify this and to delineate how the downstream residues within APIM (VK) and residues outside the APIM-motifs interact with the β-clamp. Flanking residues of both the PIP-box and APIM are shown to have a role for binding to PCNA (59,60).

### APIM-peptides inhibit DNA replication

**Figure 3.**
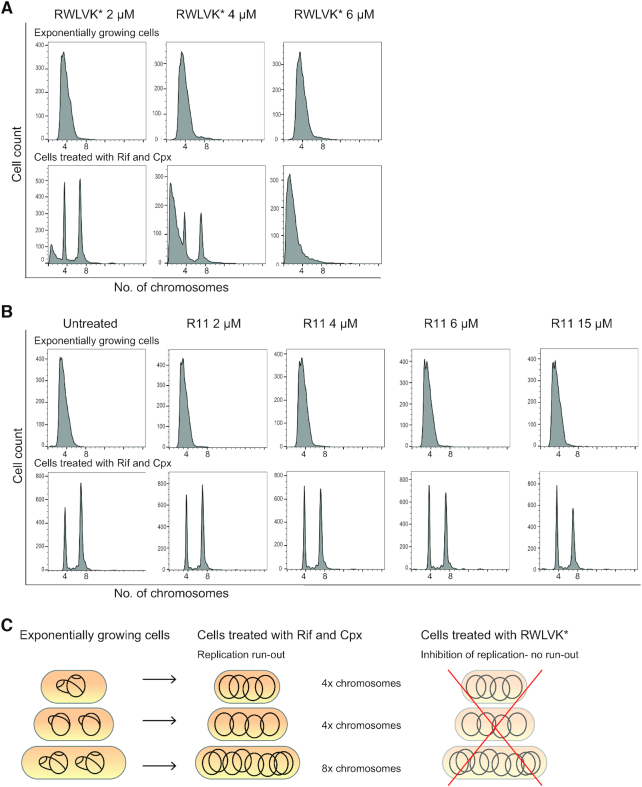
APIM-peptide variant RWLVK* blocks DNA replication in *E. coli* MG1655 cells. (**A**) Flow cytometry histograms showing the DNA content of cell penetrating RWLVK* treated cells. The top row shows the DNA histograms of cells growing exponentially, whereas the lower row shows the DNA histograms of cells treated with rifampicin (Rif) and cephalexin (Cpx) to allow for run-out of replication. The peptide concentrations (2–6 μM) are depicted in the figure. (**B**) Flow cytometry histograms showing the DNA content of untreated and R11-peptide treated cells. The top row shows the DNA histograms of cells growing exponentially, whereas the lower row shows the DNA histograms of cells treated with Rif and Cpx. The peptide concentrations (2–15 μM) are depicted in the figure. (**C**) Schematic cartoon illustrating the DNA content in actively replicating cells of exponentially growing cultures (left), the fully replicated chromosomes in cells treated with Rif and Cpx (mid), and inhibition of replication with for example RWLVK* treatment which lead to no run-out to produce four and eight chromosomes (right).

In order to directly test whether APIM-peptides inhibit the process of DNA replication we performed flow cytometry analysis of the DNA content in *E. coli* MG1655 cells treated with the cell penetrating RWLVK*-peptide and compared it to untreated cells and cells treated with the cell penetrating part only, R11. In this assay, the cells treated with Rif and Cpx cannot initiate new rounds of replication or divide but can perform run-out of already initiated replication (see ‘Materials and Methods’ section). DNA replication was partially inhibited by RWLVK* at 2 and 4 μM, and completely inhibited at 6 μM (Figure 3A, illustrated in 3C). The DNA histograms also indicated that treatment with RWLVK* initiated DNA degradation, likely as a result of double-strand breaks upon DNA replication fork arrest. In contrast, the R11 peptide did not have any effect on DNA replication under these assay conditions even at concentrations above MIC (15 μM), indicating that the cell penetrating part of the peptide has no direct effect on DNA replication (Figure 3B).

### APIM-peptides strongly inhibit mutagenesis

Because both replicative and TLS DNA polymerases bind to the same region on the β-clamp via their CBMs, the anti-mutagenic activities of the APIM-peptides, overexpressed versus added peptide in *E. coli* BL21 was next compared. Overexpression of RWLVK and RFSLK containing peptides, both of which strongly inhibited bacterial growth (Figure 1D), reduced the spontaneous mutation frequency by >98% (Table 2). Overexpression of the truncated APIM-peptide, RWLK, which did not inhibit bacterial growth nor bind to the β-clamp (Figure 1D and Supplementary Figure S2A), did not affect the mutation frequency (Table 2). The same patterns were seen by addition of cell penetrating APIM-peptides; however, overexpression reduced the mutation frequency much more than addition of the same APIM-variants (Table 2). The R11 cell penetrating peptide did not affect the mutation frequency.

**Table 2.** APIM-peptide variants reduce spontaneous mutation frequency in *E. coli*. Overexpression of RWLVK (*n* = 5), RFSLK (*n* = 5), RWLK (*n* = 6) and empty vector/control (*n* = 12) was conducted in *E. coli* BL21

	Mutation frequency (Rif^R^/10^8^ CFU)	
Peptide-variants	*Overexpressed peptides*	*Added peptides*
no peptide (control)	2.5 ± 0.4	10.5 ± 2.2
RWLVK	0.02 ± 0.01 **	5.9 ± 2.3 **
RFSLK	0.01 ± 0.003 **	4.1 ± 1.6 *
RWLK	3.8 ± 1.2	7.7 ± 3.3
R11	na	7.2 ± 3.9

Addition of cell penetrating peptide variants (20 μM) containing RWLVK (*n* = 11), RFSLK (*n* = 4), RWLK (*n* = 7), R11 only (*n* = 5) and no peptide/control (*n* = 11) was conducted in *E*. *coli* MG1655. Average ± SEM, *n* = independent biological experiments. Two tailed, paired *t*-test, P < 0.05*, P ≤ 0.005**.

The strong reduction in Rif^R^ colonies detected when overexpressing peptides compared to adding peptides might be exaggerated due to strong inhibition of growth. Additionally, the intracellular levels of the APIM-peptides are likely much lower when added as a cell penetrating peptide compared to when continuously overexpressed. Another contributing factor may be different APIM-flanking regions in the overexpressed peptides (GILQSTLA) compared to the added cell penetrating RWLVK and RFSLK-peptides (WKKKRKI) used in these experiments. Flanking regions could influence import, the stability of the peptides (both in the media and inside the bacteria), and/or interactions with the β-clamp as well as contribute to negative selection as seen for PCNA ligands (60).

**Figure 4.**
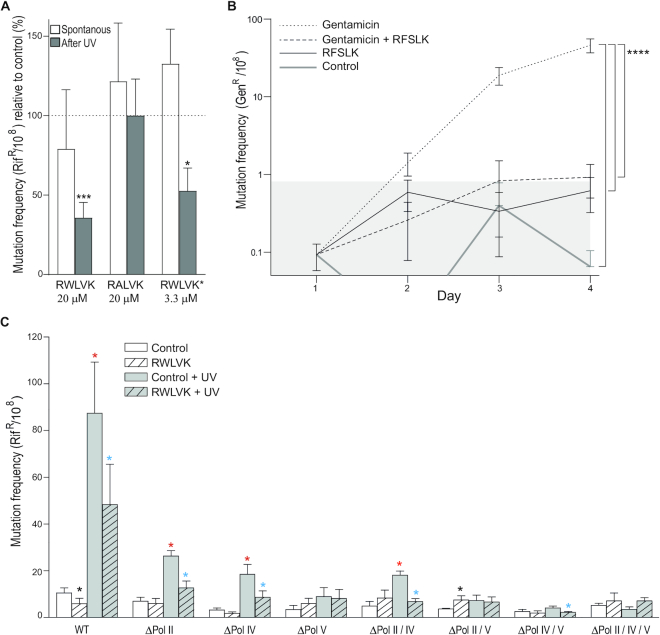
APIM-peptides inhibit TLS. (**A**) Spontaneous and UV induced (2 mJ/cm^2^) mutation frequency in *E. coli* MG1655 cells after the addition of the cell penetrating RWLVK or RALVK-peptides (20 μM), *n* = 8 and *n* = 7 respectively, or RWLVK*-peptide (3.3 μM), *n* = 7. The bars represent fraction of rifampicin resistant CFU (Rif^R^/10^8^) of *n* = independent biological experiments relative to control (%) ± SEM. Control = no peptide added, represented by the dotted line. Two-tailed, paired *t*-test against control, *P* < 0.05*, *P* < 0.001***. (**B**) Mutation frequency in a gentamicin sensitive clinical isolate of *Staphylococcus epidermidis* after incubation with gentamicin (0.25 μg/μl) and/or the cell penetrating RFSLK APIM-peptide (3 μM) for 4 days. Control = no treatment. Mutation frequency is shown as average gentamicin resistant CFU (Gen^R^/10^8^) from *n*≥ 4 independent biological experiments ± SEM, gray area = mutation frequency ± SEM of the control culture. *P* < 0.0001****, two-way ANOV A. (**C**) Spontaneous and UV induced (2 mJ/cm^2^) mutation frequency in *E. coli* MG1655 in WT and TLS deletion mutant cells after addition of the cell penetrating RWLVK-peptide (20 μM). Spontaneous mutation frequency from untreated cultures (white bars, control) and after addition of APIM-peptide (white bars with a shaded pattern, RWLVK). Mutation frequency in UV-irradiated cultures without treatment (gray bars, control + UV) and after addition of APIM-peptide (gray bars with a shaded pattern, RWLVK + UV). Black* = significant change from untreated culture after RWLVK-peptide addition, Red* = significantly increased mutation frequency from untreated cultures after UV-irradiation. Blue* = significantly reduced mutation frequency after addition of RWLVK-peptide to UV-irradiated cultures. Average mutation frequency (Rif^R^/10^8^) ± SEM, *n* = 8–10, *n* = independent biological experiments. Two-tailed, paired *t*-test relative to WT control, *P* < 0.05*.

Next, the ability of the cell penetrating APIM-peptides to reduce UV-induced mutation frequency was examined. RWLVK, which binds to the β-clamp, but not the much weaker β-clamp binder, RALVK, reduced the mutation frequency (Figure 4A). This further supports that the RWLVK peptide bind to the same site on the β-clamp as the bacterial TLS polymerases, and that the affinities of the APIM-variants for the β-clamp correlated with their ability to inhibit mutagenesis. In support for the involvement of flanking regions, reduced MIC (Figure 1C) and more efficient reduction of the mutation frequency; a 53% reduction using only 1/6 of the molar concentration was found for the RWLVK-peptide containing GILQ-WRKI (RWLVK*) instead of WKKK-RKI (i.e. RWLVK) in front of the cell penetrating peptide sequence (Figure 4A). These results support that the APIM-flanking regions contribute to the antibacterial and anti-mutagenic activities of the peptides.

To examine the anti-mutagenic effects of cell penetrating APIM-peptides during antibiotic-induced stress in continuously growing cultures, *S. epidermidis* was grown in sub-MIC levels of gentamicin with and without the RFSLK-peptide and the fractions of gentamicin resistant (Gen^R^) bacteria were determined. Several antibiotics are shown to induce SOS in *S. aureus* (61,62), and sub-lethal concentrations of gentamicin is shown to induce SOS in *E. coli* (63). The bacterial cultures were treated on day 1 and grown for three additional days with daily re-inoculation (1:100) in media containing fresh drugs. The cultures that were grown with a low dose of only gentamicin quickly (after 2 days) developed a statistically significant fraction of gentamicin resistance bacteria compared to those grown in cultures with the combination of gentamicin and the RFSLK-peptide (Figure 4B). RFSLK-peptide addition alone did not change the mutation frequency compared to the control. This indicates that APIM-peptides used in combination with gentamicin can inhibit resistance development and supports that the APIM-peptides inhibits TLS.

### APIM-peptides mainly inhibit Pol V-mediated TLS

To investigate the role of the individual TLS polymerases and the anti-mutagenic activity of the cell penetrating RWLVK APIM-peptide after induction of SOS by UV-irradiation, we made single, double and triple TLS polymerase deletion mutants in *E. coli* MG1655. The RWLVK-peptide significantly reduced the spontaneous mutation frequency in wild type (WT), but not in any of the TLS deletion strains (Figure 4C, black *). Interestingly, increased mutation frequency was detected in the ΔPol II/V strain, suggesting that the RWLVK-peptide has a larger capacity for inhibiting the binding of Pol III to the β-clamp than Pol IV under these conditions. Inducing SOS with UV-irradiation increased the mutation frequency in all the Pol V proficient strains (Figure 4C, red *), in accordance with Pol V being the main TLS polymerase for bypass of UV-damages (64). Treatment with the RWLVK-peptide reduced the mutation frequency in all Pol V containing strains after UV-damage (Figure 4C, blue *). In the ΔPol IV/V strain we could not detect any significant increase in mutation frequency after UV-irradiation; still, we detected a reduction in the mutation frequency after RWLVK-peptide treatment. This may indicate an increased affinity of RWLVK for the β-clamp after SOS similarly to what is seen for the APIM-PCNA interaction upon DNA damage and poly-ubiquitination (22). This could be mediated by modifications on the β-clamp and/or other accessory proteins and could potentially explain the low *in vitro* affinity of the peptides for purified β-clamp compared to the high capacity for inhibiting replication and TLS *in vivo*. Strains lacking Pol II grew slower and were also less inhibited by the APIM-peptide than the other strains, both under untreated conditions and after SOS induction (Supplementary Figure S4), supporting that replication is targeted by the APIM-peptide. Taken together, these results suggest that the APIM-peptide can inhibit the interaction of Pol II and Pol V with the β-clamp, and that this is more pronounced after SOS induction (UV). No reduction of mutation frequency was detected in the TLS deficient ΔPol II/IV/V strain by APIM-peptide addition under any conditions. When examining the mutation spectra in the *rpoB* gene we found that the RWLVK-peptide modulated the pattern of mutations; however, no clear trends in the mutation spectra from the different strains could be extracted (data not shown).

In summary, these results suggest that APIM-peptides compete with the TLS polymerases for binding to the β-clamp. This further suggests that the region harboring subsite I and subsite II in the C-terminal part of the β-clamp is capable of interacting with a wide range of peptides diverging from the rather broadly defined CBM, as well as clamp binding motifs from eukaryotes. This region in PCNA is also promiscuous and can bind to a wide range of PIP-box and APIM-variants (11,60,65).

### *In vivo* proof of concept; APIM-peptides strongly reduce the growth of MRSA in a skin infection model in mice

**Figure 5.**
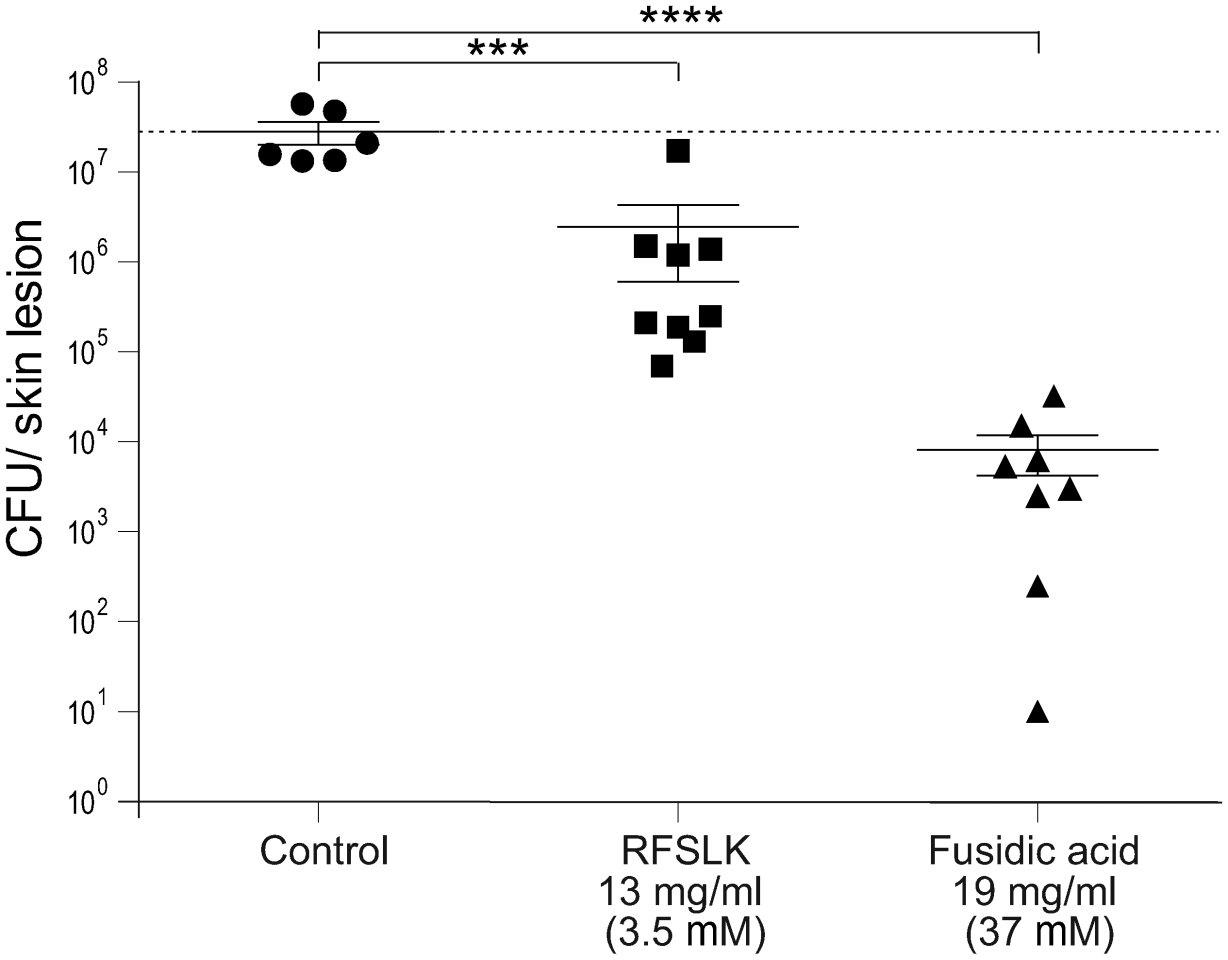
APIM-peptides significantly reduce growth in a superficial skin infection model in mice. Growth of MRSA 43484 in mouse skin lesions (CFU/skin lesion) on day 4. Skin lesions were infected on day 0 and treated topically twice per day with the cell penetrating RFSLK-APIM peptide (13 mg/ml, 3.5 mM) or fusidic acid (19 mg/ml, 37 mM) on day 1–3. No treatment = control. The dashed line represents the mean of inoculum (CFU/skin lesion) from animals sacrificed on day 1, before treatment start. *P* < 0.001***, *P* < 0.0001****, ANOVA, multiple comparisons.

At last, as an *in vivo* proof of concept we tested the efficacy of the cell penetrating RFSLK APIM-peptide in a murine skin infection model. Superficial skin wounds on the back of mice was infected with MRSA and treated topically with a methyl cellulose gel containing APIM-peptide twice a day for 3 days. We found that the APIM-peptide gel significantly reduced the bacterial load in the infected lesions compared to controls only treated with the empty gel (Figure 5). High efficacy was achieved even though this pilot gel formulation was not optimal, being of much lower viscosity than the positive control (fusidic acid ointment). However, and more importantly, no toxicity of the APIM-peptide gel was detected. Although we cannot directly compare the activity of the APIM-peptide with fusidic acid in this experiment, a significant reduction in bacterial load was detected at a 10-fold lower molar concentration of the APIM-peptide than fusidic acid, showing great promise for further development.

### Concluding remarks

Based on the observed similarity in polymerase binding sites between PCNA and the β-clamp, published data and our results, we find that peptides containing APIM bind to both PCNA and the β-clamp. The APIM-peptides inhibit bacterial growth at lower concentrations than needed for growth inhibition of the most sensitive cancer cells (10). The direct interaction with the β-clamp likely mediates parts of the APIM-peptides’ growth inhibition and all their anti-mutagenic properties. APIM-peptides have anti-mutagenic activities at doses below MICs, suggesting that the TLS Pol II and Pol V are efficiently inhibited at concentrations not completely blocking Pol III mediated replication. To our knowledge, no other specific inhibitor(s) of bacterial TLS polymerases/processes has been developed; thus, the APIM-peptide's ability to reduce mutagenesis might have great potential in the battle against the development of antibiotic resistance if used in combination with other antibiotics. *In vivo* proof of concept in a murine skin infection model supports further development of these peptides.

## Supplementary Material

gkaa278_Supplemental_File

## Notes

**Disclaimer:** The funders had no role in the study design, data collection and analysis, decision to publish, or preparation of the manuscript.
