# Supplementary material for: Peptides containing the PCNA interacting motif APIM bind to the β-clamp and inhibit bacterial growth and mutagenesis
Source: Nucleic Acids Res. 2020 Apr 29;48(10):5540–54. doi: 10.1093/nar/gkaa278 (PMC7261172; doi:10.1093/nar/gkaa278)
Supplement: gkaa278_Supplemental_File [file gkaa278_supplemental_file.pdf]

Supplementary data

Peptides containing the PCNA interacting motif APIM bind to the β-clamp and inhibit bacterial growth and mutagenesis

Aina Nedal[1, #], Synnøve Brandt Ræder[1, #], Bjørn Dalhus[2,3], Emily Helgesen[1,3], Rune Johansen Forstrøm[3], Kim Lindland[1], Balagra Kasim Sumabe[1], Jacob H. Martinsen[4], Birthe B. Kragelund[4], Kirsten Skarstad[3], Magnar Bjørås[1,3] and Marit Otterlei[1*]

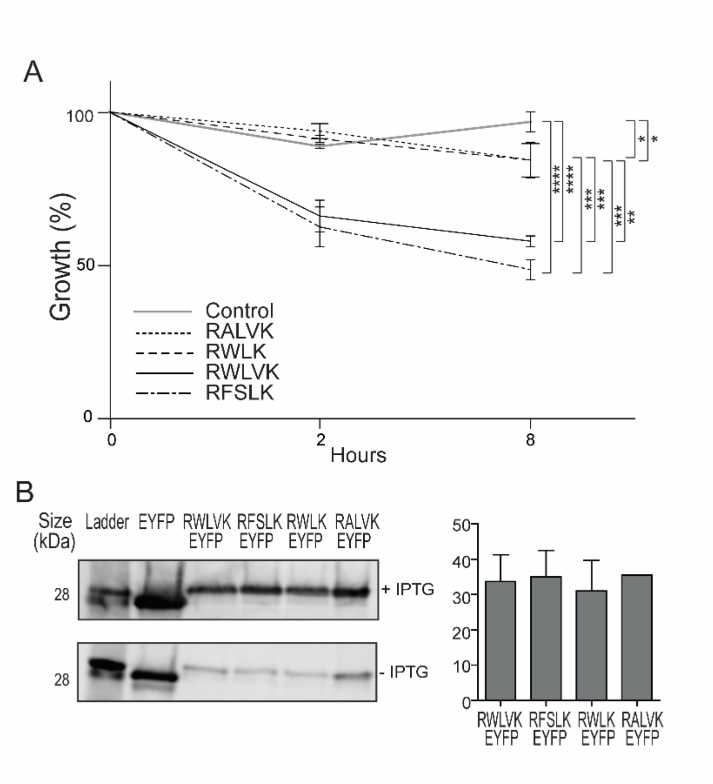

Supplementary Figure S1: Additional growth measurements (OD_{600}) of BL21 expressing different APIM variants and the quantification of the expression of the peptides.

(**A**) Effect of overexpressing different APIM-peptides (RWLVK, RFSLK, RWLK and RALVK) on BL21 growth. Control= BL21 without vector. Experiments are performed as described in Figure 1D. Data presented represent average data from 3 technical replicas from 4 biological experiments (3 different bacteria clones for each peptide variant) p<0.0001****, p<0.001***, p<0.01**, p<0.05*, two-way ANOVA. (**B**) The expression level of different APIM-variants (RWLVK, RFSLK, RWLK and RALVK) four hours after induction with IPTG. WB showing cell extracts from cultures with and without IPTG induction (upper and lower panel respectively). APIM-peptides are expressed as a fusion peptide with EYFP expressed from pET-28a(+) vector. A vector expressing only EYFP is used as a control. Quantification (right panel) is based on EYFP expression (αGFP antibody ab290, Abcam), normalized against control. Average of measurements from three membranes are shown (except RALVK-EYFP where only two membranes are included).

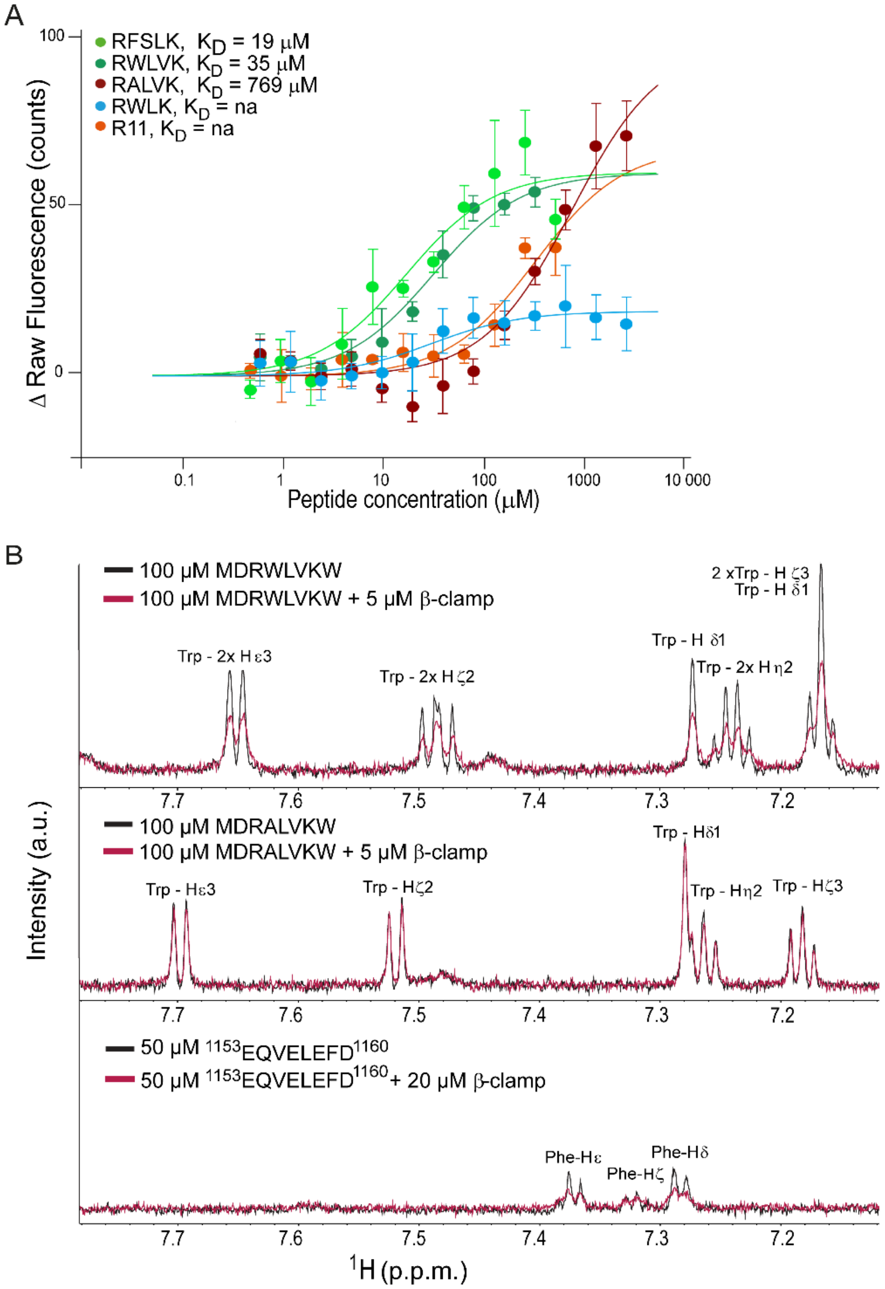

Supplementary Figure S2: Binding measurement indicate a direct interaction between APIM and the β-clamp.

(**A**) Microscale thermophoresis measurements (ΔRaw fluorescence) and dissociation constants for interaction between the *E. coli* β-clamp and full-length cell-penetrating APIM-peptides (Ac-MD-"**APIM"**-W-KKKRK-I-R11), where "APIM" is: **RWLVK**, K_D=35 μM, **RFSLK** K_D=19 μM and **RALVK** K_D=769 μM. K_Ds for **RWLK** (truncated APIM) and R11 were not applicable. Curves are based on 3 independent experiments (n=3, mean ± STD). (**B**) 1D ^{1}H NMR spectra (aromatic region) of the RWLVK (top) alone (black, 100 μM) and with β-clamp (red, 1 μM), the RALVK alone (black, 100 μM) and with β-clamp (red, 1 μM) and Pol III binding peptide (^{1153}E<u>QVELEF</u>D^{1160})(bottom) alone (black, 50 μM) and with β-clamp (red, 20 μM). The NMR spectra were recorded at 298 K with nt=32 and the assignment of peaks are indicated.

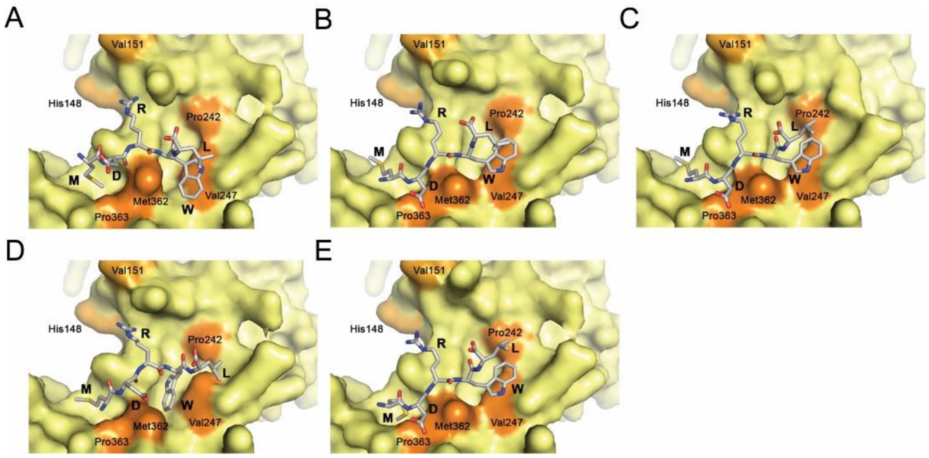

Supplementary Figure S3. Top 5 docking models from Rosetta FlexPepDock using the crystal structure of Pol V peptide binding to β-clamp as input, with residues QLNLF replaced by MDRWL.

In model (**A-C**) and (**E**) the W-residue of APIM is interacting with the V247 residue in β-clamp, while in one model (**D**), the W-residue is wedged in-between V247 and M362.

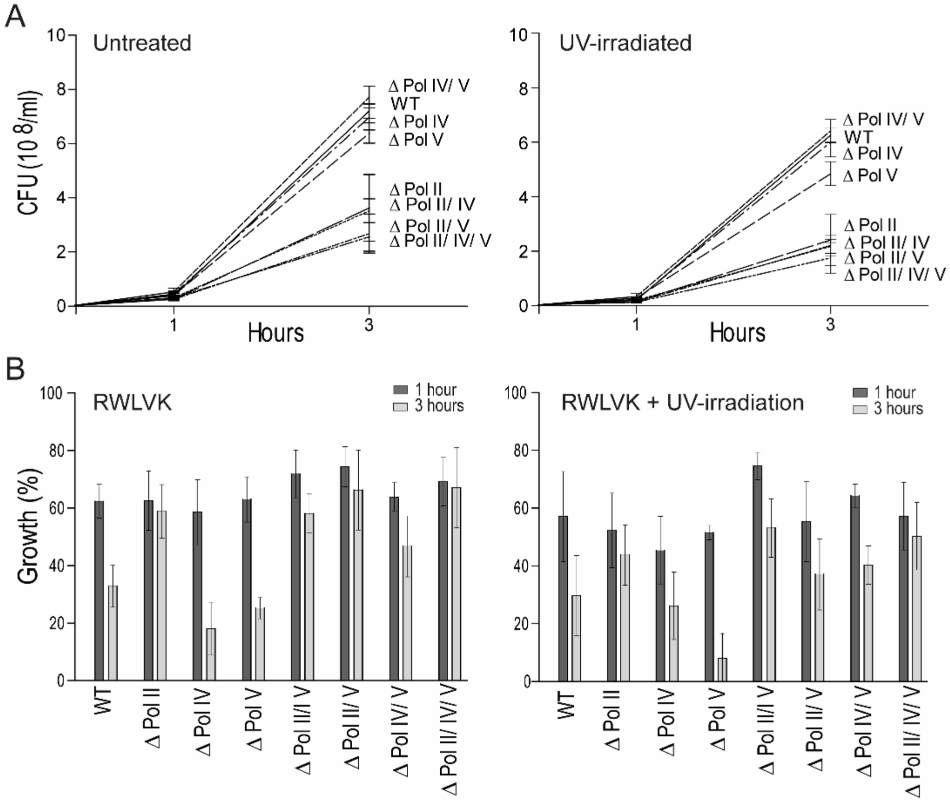

Supplementary Figure S4: Growth and sensitivity of the single, double, and triple TLS polymerase deletion strains.

(**A**) Growth (CFU) of *E. coli* MG1655 WT and single, double and triple TLS polymerase deletion (ΔPol II, ΔPol IV, ΔPol V, ΔPol II/IV, ΔPol II/V, ΔPol IV/V and ΔPol II/IV/V) cultured under normal conditions (untreated,

left panel) and after UV-irradiation (right panel). (**B**) Growth of WT and the deletion mutants relative to untreated cultures (%) 1 and 3 hours after the addition of the cell-penetrating APIM-peptide, RWLVK (20 µM). Left: cultured under normal conditions, right: UV-irradiated cultures. Mean ± SEM, n=5 independent biological experiments.
